# Upregulation of C-X-C motif chemokine 12 in the spinal cord alleviated the symptoms of experimental autoimmune encephalomyelitis in Lewis rats

**DOI:** 10.3389/fnins.2023.1105530

**Published:** 2023-03-16

**Authors:** Dahe Lin, Hongjuan Liu, Honglu Song, Biyue Chen, Junxia Fu, Mingming Sun, Huanfen Zhou, Wenhao Bai, Shihui Wei, Hongen Li

**Affiliations:** ^1^Department of Ophthalmology, The First Medical Center of Chinese People’s Liberation Army (PLA) General Hospital, Beijing, China; ^2^Fujian Provincial Key Laboratory of Ecology-Toxicological Effects and Control for Emerging Contaminants, College of Environmental and Biological Engineering, Putian University, Putian, Fujian, China; ^3^Key Laboratory of Ecological Environment and Information Atlas, Fujian Provincial University (Putian University), Putian, Fujian, China; ^4^Department of Ophthalmology, Beijing Tongren Eye Center, Beijing Tongren Hospital, Beijing, China; ^5^Department of Ophthalmology, The 980th Hospital of the Chinese People’s Liberation Army (PLA) Joint Logistics Support Force, Shijiazhuang, Hebei, China; ^6^Department of Ophthalmology, The Third Medical Center of Chinese People’s Liberation Army (PLA) General Hospital, Beijing, China

**Keywords:** CXCL12, EAE, AAV, neuroinflammation, remyelination

## Abstract

**Background:**

C-X-C motif chemokine 12 (CXCL12) is a chemokine that performs many functions. Studies have shown that CXCL12 can aggravate inflammatory symptoms in the central nervous system (CNS). Evidence also indicates that CXCL12 can promote the repair of myelin sheaths in the CNS in experimental autoimmune encephalomyelitis (EAE). Here, we investigated the function of CXCL12 in CNS inflammation by upregulating CXCL12 in the spinal cord and subsequently inducing EAE.

**Materials and methods:**

CXCL12 upregulation in the spinal cords of Lewis rats was induced by the injection of adeno-associated virus 9 (AAV9)/eGFP-P2A-CXCL12 after intrathecal catheter implantation. Twenty-one days after AAV injection, EAE was induced and clinical score was collected; Immunofluorescence staining, WB and LFB-PAS staining were used to evaluate the effect of CXCL12 upregulation. In the *in vitro* study, oligodendrocyte precursor cells (OPCs) were harvested, cultured with CXCL12 and AMD3100, and subjected to immunofluorescence staining for functional assessment.

**Results:**

CXCL12 was upregulated in the lumbar enlargement of the spinal cord by AAV injection. In each stage of EAE, upregulation of CXCL12 significantly alleviated clinical scores by inhibiting leukocyte infiltration and promoting remyelination. In contrast, the addition of AMD3100, which is a CXCR4 antagonist, inhibited the effect of CXCL12. *In vitro*, 10 ng/ml CXCL12 promoted the differentiation of OPCs into oligodendrocytes.

**Conclusion:**

AAV-mediated upregulation of CXCL12 in the CNS can alleviate the clinical signs and symptoms of EAE and significantly decrease the infiltration of leukocytes in the peak stage of EAE. CXCL12 can promote the maturation and differentiation of OPCs into oligodendrocytes *in vitro*. These data indicate that CXCL12 effectively promotes remyelination in the spinal cord and decreases the signs and symptoms of EAE.

## Introduction

As a common manifestation of multiple sclerosis (MS) and neuromyelitis optica spectrum disorders (NMOSDs), inflammatory demyelination make an important contribution to disability in the patients with demyelinating disorders (Kearney et al., 2015; Ciccarelli et al., 2019; Fadda et al., 2022). Chemokines are considered to be essential mediators that are secreted by many kinds of cells to activate G protein-mediated signaling pathways (Krumbholz et al., 2006; Huynh et al., 2020). Some of the chemokines guided inflammatory cells into the central nervous system (CNS) and played an important role in the myelin damage, but some are not, Notably, chemokine C-X-C motif ligand 12 (CXCL12) was revealed to play a crucial role in the maintenance of neural homeostasis, including the regulation of proliferation, differentiation and migration of oligodendrocyte precursor cells (OPCs) in the animal disease model system (Patel et al., 2010, 2012; Li et al., 2012; Zilkha-Falb et al., 2016). Additionally, CXCL12 could recruit other types of endogenous stem/progenitor cells, such as hematopoietic stem cells, mesenchymal stem cells, endothelial progenitor cells and neural progenitor cells (NPCs), mainly worked by interacting with CXCR4, which is one of its natural receptors (Klein and Rubin, 2004; Dziembowska et al., 2005; Patel et al., 2010; Carbajal et al., 2011; Huynh et al., 2020).

The observation that up-regulation of CXCL12 were found in the reactive astrocytes and endothelial cells in the lesions of patients with MS, and CXCR4-positive leukocytes could further infiltrated into CNS parenchyma (Calderon et al., 2006; McCandless et al., 2008; Moll et al., 2009). McCandless et al. (2008) suggested that the redistribution of CXCL12 at the blood-brain barrier (BBB), rather than up-regulation of CXCL12 expression in lesions of CNS, plays a more important role in leukocyte infiltration and demyelination in MS. These phenomenon are also observed in Experimental Autoimmune Encephalomyelitis (EAE), which may be the most frequently used animal model system of rodent for MS studying (McCandless et al., 2006; Cruz-Orengo et al., 2011; Zilkha-Falb et al., 2016). Inhibition of CXCL12 signaling could result in widespread white matter infiltration of mononuclear cells and aggravate EAE symptoms (McCandless et al., 2006). Therefore, it is rational to speculate that the ongoing progression of MS may result from the enhanced infiltration of leukocytes.

A series of studies have examined EAE in Dark Agouti (DA) and Albino Oxford (AO) rats (Miljković et al., 2011; Blaževski et al., 2013, 2015). The differential resistance of these two rat strains to EAE implies that CXCL12 is involved in the promotion of remyelination (Miljković et al., 2011; Blaževski et al., 2013). Many of excellent studies have shown that CXCL12 plays a positive role in promoting the remyelination of EAE (McCandless et al., 2006; Patel et al., 2010, 2012; Miljković et al., 2011; Blaževski et al., 2013; Zilkha-Falb et al., 2016). The intense inflammation and macrophage phagocytosis aggregation in the active MS lesions are the main causes that leads to failure of remyelination, since sufficient OPCs were found within lesions (Kuhlmann et al., 2008; Lassmann et al., 2012; Chu et al., 2017). On the basis of the function of CXCL12 that promote migration and differentiation of OPCs, upregulation of CXCL12 in CNS may be a potential therapeutic strategy to MS. Several previous studies showed that CXCL12 acts as a T-cell chemoattractant at low doses and a chemorepellent at high doses (Poznansky et al., 2000; Vianello et al., 2006). So it is interesting to up-regulate CXCL12 before the onset of EAE, and observe whether the upregulated CXCL12 can alleviate the symptoms, or even inhibit the occurrence of EAE.

In this study, polyethylene catheters (PE-10 tubing) were placed into the subarachnoid space near the lumbar enlargement of rats, and overexpression of CXCL12-GFP in this area of CNS was induced by injection of AAV through the PE-10 tubing. 21 days after infection, EAE model was builded. The aim was to verify whether direct upregulation of CXCL12 gene in the spinal cord could promote the remyelination or exacerbate EAE symptoms by enhancing leukocyte infiltration.

## Materials and methods

### Animals and intrathecal catheter implantation

Ten-week-old female Lewis rats (180–220 g) were purchased from Vital river of China and raised under specific pathogen-free conditions. The rats were anesthetized with sodium pentobarbital (50 mg/kg), and then a polyethylene catheter was inserted through a hole made in the posterior atlantooccipital membrane (Chen et al., 2020). The catheter was threaded 7 cm caudally into the subarachnoid space of the spinal cord (Chen et al., 2020). The end of the tube opened approximately in the lumbar enlargement. The rostral part of the tube was sutured to the muscle for immobilization. After intrathecal catheter implantation, the rats were housed individually and allowed to recover for 7 days before AAV9 was injected into the subarachnoid space through a PE-10 tube (Portex Tubing PE 0.28*0.165 mm Bx, Scientific Laboratory Supplies, Nottingham).

### AAV vector production

The AAV9 vectors (pAAV-CMV-bGlobin-EGFP-P2A) were used in this study to package rat CXCL12 (GenBank ID: NM_022177). AAV9 viral stocks with the recombinant AAV vector were produced according to the three-plasmid cotransfection method by Obio Technology Corp., Ltd., (Shanghai, China) (Beckman et al., 2021). The titers of purified AAV9/eGFP and AAV9/eGFP-P2A-CXCL12 were measured by quantitative polymerase chain reaction (qPCR) using SYBR green technology.^1^ The AAVs were aliquoted and stored at −80°C until further use.

### Study design

To determine the functions of CXCL12 in the rat CNS in EAE, an AAV9 vector expressing CXCL12 (AAV9/eGFP-P2A-CXCL12) was administered, as mentioned above; an eGFP vector (AAV9/eGFP) acted as the control. Each rat received 1 × 10^12^ vg/ml AAV9/eGFP-P2A-CXCL12 or AAV9/eGFP intrathecally *via* a polyethylene catheter that was inserted through a small hole made in the posterior atlantooccipital membrane. Then, the rats were divided into three groups: CXCL12, eGFP and CXCL12 + AMD3100. AMD3100, a CXCR4 antagonist, was injected into the CNS through the catheter 2 days after immunization with spinal cord homogenate (SCH) and complete Freund’s adjuvant (CFA). AMD3100 (Sigma-Aldrich, St. Louis, MO, USA), was dissolved in normal saline to an injection dose of 40 μg/10 μl per rat.

### Induction of EAE

Twenty-one days after AAV injection, the rats were immunized with a 400-μl mixture containing 200 μl of spinal cord homogenate (SCH) and an equal volume of complete freund’s adjuvant (CFA, Sigma) by subcutaneous injection in the base of the tail. Forty-eight hours after immunization, the rats received 400 ng of pertussis toxin (Sigma) in 200 μl of PBS *via* intraperitoneal injection. The animals were observed and scored. Scoring was completed using a standard five-point scale: 0, no deficit; 0.5, partial loss of tail tone or slightly abnormal gait; 1.0, complete tail paralysis or both partial loss of tail tone and mild hind limb weakness; 1.5, complete tail paralysis and mild hind limb weakness; 2.0, tail paralysis with moderate hind limb weakness (evidenced by frequent foot dragging); 2.5, no weight bearing on hind limbs (dragging) but with some leg movement; 3.0, complete hind limb paralysis with no residual movement; 3.5, hind limb paralysis with mild weakness in forelimbs; 4.0, complete quadriplegia but with some movement of the head; 4.5, moribund; and 5.0, death.

### Histological and fluorescent immunostaining

First, the rats were anesthetized by intraperitoneal injection of pentobarbital sodium (50 mg/kg), and then the rats were perfused through heart with precooled PBS followed by precooled 4% paraformaldehyde (PFA) in PBS. The spinal cord was isolated and fixed for 6 h and then immersed in 30% sucrose for 48 h. After freezing in Tissue–Tek OCT compound (Sakura, Japan), the spinal cord was sliced by a freezing microtome (CM1850, Leica Biosystems, Heidelberg, Germany); 10-μm-thick coronal sections were prepared and processed for Luxol fast blue periodic acid-Schiff (LFB-PAS) (Sigma-Aldrich, St. Louis, MO, USA) staining as well as fluorescent immunostaining for detect the severity of demyelination in each stages. The slides were dehydrated to 95% alcohol and incubated in 0.1% LFB solution about 10–16 h at 58°C, then sections were differentiated in 0.05% lithium carbonate solution and 70% alcohol then counterstained with PAS (Li et al., 2013). For fluorescent immunostaining, the slides were washed with PBS, treated with blocking solution (5% normal donkey serum in PBS) for 1 h at room temperature (25°C) and then stained with one of the following primary antibodies (diluted in blocking solution) in a humidified box overnight at 4°C: anti-MBP (1:100, Abcam, UK); anti-GFAP (1:200, CST, USA); anti-NG2 (1:100, Abcam, UK); anti-CD45 (1:100, Abcam, UK); anti-CXCR4 (1:100, Abcam, UK); and anti-CXCL12 (1:100, Abcam, UK). Finally, the slides were exposed to the appropriate secondary antibodies (1:200, Abcam, UK) for 1 h in a humidified box at room temperature. After immunostaining, the slides were stained with DAPI to visualize the nuclei.

### Western blot analysis

The spinal cord tissue of Lewis rats about 0.5 cm upstream and downstream of the PE-10 tube’s end was obtained, and then cut the tissue into 1–2 mm^3^ small pieces with ophthalmic scissors. After adding 3–5 times the volume of RIPA lysis buffer (Beyotime, China) and resuspending, the tissue was homogenized and then gets ultrasonication for 30 s. Centrifuged the homogenate at 14,000 *g* 4°C for 10 min for the supernatant and detected the protein concentration by the BCA kit (Beyotime, China). Diluted the sample protein concentration to 2 μg/μl with RIPA lysis buffer. The protein samples (30 μg/each) denatured in 5x loading buffer at 100°C for 5 min. Samples were loaded and separated electrophoretically using 8% SDS-PAGE gel operated at 100 V (for 1 h) and then 130 V (for 1.5 h), respectively. Following electrophoresis, samples were transferred to PVDF membranes (invitrogen, USA) using transfer buffer contain 25 mM Trisbase, 192 mM Glycine and 20% methanol. Membranes were blocked with 5% skimmed milk in Tris–buffered saline/0.1% Tween (TBST) for 1 h at room temperature, followed by incubation with primary antibody (rabbit anti-CXCL12, 1:1000, Abcam, UK; rabbit anti-GFP, 1:1000, Abcam, UK; mouse anti-β-actin, 1:1000, Abcam, UK) in blocking buffer (3%BSA in TBST) for 16 h at 4°C. Membranes were washed 3 × 15 min in TBST, and then incubated with goat anti-rabbit or goat anti-mouse IgG (H + L) secondary antibodies conjugated to horse-radish peroxidase (1:1000, Beyotime, China) for 1 h at room temperature, washed 3 × 15 min in TBST, and then visualized with ECL substrate (Thermo Scientific, USA) and imaged with ChemiDoc MP system (Bio-Rad, USA).

### Culture of OPCs

Oligodendrocyte precursor cells (OPCs) of WISTAR rats were purchased from CHI Scientific Co., Ltd., (1-5110). OPCs were cultured in Dulbecco’s modified Eagle’s medium (DMEM):F12 media (Gibco company, USA) supplemented with HEPES (Sigma, USA), bFGF (human recombinant, 20 ng/ml, PeproTech, USA) and EGF (mouse recombinant, 20 ng/ml, PeproTech, USA), all from PeproTech Company, USA. The culture medium was renewed every 2 days. For the differentiation experiments, OPCs were cultured in the presence or absence of 10 ng/ml CXCL12 (PeproTech, USA) in differentiation medium. To demonstrate the mechanism underlying the promotion of OPC differentiation, cells were treated with AMD3100 (100 ng/ml) and incubated for 12 days in differentiation medium. AMD3100 medium was replaced every day.

### Image analysis

All images were captured using a Leica DM4000B microscope. The software settings for imaging kept exactly identical among spinal cord sections in each immunostaining. Areas of demyelination in the lumbar enlargement of spinal cords were quantified using a 0–4 points semiquantitative scale system (Zhang et al., 2019), where 0 = no demyelination; 1 = rare and focal demyelination; 2 = multiple focal demyelination; 3 = large or confluent demyelination; 4 = large and confluent demyelination. All slides were read in a blinded manner. The fluorescence intensity was calculated as the percentage of the antibody (anti-MBP, anti-GFAP, anti-NG2, and anti-CD45) positive stained area to the total area of spinal cord in the figure, and results from each rat were counted in three slides. Statistical analysis was undertaken using ImageJ software (version 1.39, NIH, USA).

### Data statistics

The data were expressed as mean ± SEM (standard error of mean). 1-way ANOVA (analysis of variance) was used to test significant differences among three groups, and 2-tailed unpaired Student’s *t*-test was used for two groups at each time point. *P* < 0.05 was considered statistically significant.

## Results

### Upregulation of CXCL12 in the spinal cord lumbar nlargement of Lewis rats

**FIGURE 1:**
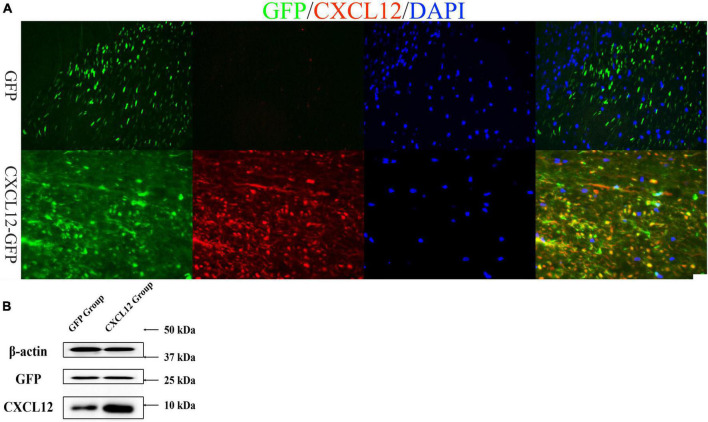
CXCL12-GFP and eGFP were upregulated in the lumbar enlargement of the spinal cord. **(A)** Adeno-associated virus (AAV)-mediated upregulation of CXCL12 and eGFP in the lumbar enlargement was robust. On the 21st day after infection, immunofluorescence showed that the level of CXCL12 in the lumbar enlargement was much higher in the CXCL12-GFP group than in the eGFP group, and the CXCL12 and eGFP signals in the white matter were stronger than those in the gray matter of the spinal cord. The bar in the lower right represents 50 μm. **(B)** Western blot (WB) results also show that the expression of CXCL12 in spinal cord of CXCL12 group, particularly in regions surrounding the cannula tip, was higher than the eGFP group.

After 21 days of the intrathecal injection of AAV9 (CXCL12 or eGFP), rats were sacrificed for immunofluorescence analysis and Western blot (WB) tests (Figure 1). We found green fluorescence in both groups (CXCL12 and eGFP) in the lumbar enlargement of the spinal cord in Lewis rats (Figure 1A). The signal of CXCL12 were significantly higher in the CXCL12 group than in the control group (eGFP group) (Figure 1A), and WB of spinal cord tissue homogenates confirmed that (Figure 1B).

### Upregulation of CXCL12 in the CNS can alleviate EAE clinical scores

**FIGURE 2:**
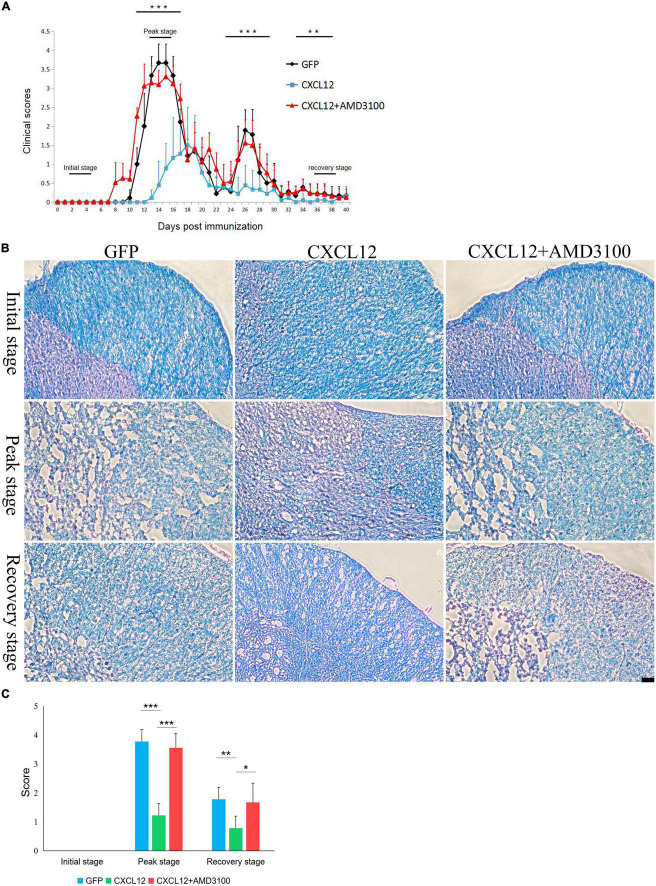
Upregulation of CXCL12 in the spinal cord can effectively reduce the clinical scores and demyelination of experimental autoimmune encephalomyelitis (EAE). **(A)** EAE was induced in Lewis rats (*n* = 15 in each group). The clinical scores were determined as described in the Methods section. The horizontal bars show the stages of EAE progression (the initial stage was 2–4 days, the peak stage was 12–14 days, and the recovery stage was 38–40 days). The rats were sacrificed for immunofluorescence detection at the each stage. **(B)** Representative images of Luxol fast blue periodic acid-Schiff (LFB-PAS) staining in the spinal cord at three stages in all three groups. The bar in the lower right represents 50 μm. **(C)** Demyelination score based on LFB-PAS staining in spinal cord. Data presented as the mean ± SEM. “*” represents value of *P* < 0.05, “**” represents value of *P* < 0.01, “***” represents value of *P* < 0.001 by 2-tailed unpaired Student’s *t*-test or 1-way ANOVA.

Experimental autoimmune encephalomyelitis (EAE) was induced in the three groups: the eGFP group, CXCL12 group and CXCL12 + AMD3100-treated group. Each group contained nine rats. The clinical scores of all rats were collected and then statistically analyzed. The results indicated that the EAE symptoms of the AMD3100 treatment group were observed the earliest (8th day), followed by the eGFP group (10th day) and the CXCL12 group (13th day) (Figure 2A). At the peak stage of EAE (12–14 days), the mean of clinical scores of the CXCL12 group were significantly lower than that of the other two groups, and no relapse of EAE was observed in the CXCL12 group between the 24th and 30th day after EAE induction (Figure 2A). In addition, at the recovery stage (33–38 days), the clinical scores of CXCL12 group were still significantly lower than that of the other two groups (Figure 2A). Daily injection of 40 μg of AMD3100 after induction of EAE through a PE-10 tube inserted in the back of the head caused earlier onset of symptoms and extended the duration of remyelination. The LFB-PAS staining showed that there were no difference among three groups at the initial stage (2–4 days) of EAE, however, higher demyelination scores of eGFP and CXCL12 + AMD3100 groups compare to CXCL12 group in the spinal cord at the peak stage and the recovery stage (Figures 2B, C). The above results indicate that upregulation of CXCL12 in the white matter of the spinal cord can effectively reduce clinical scores and alleviate symptoms in the EAE model. Moreover, upregulated CXCL12 appeared to inhibit the recurrence of EAE (Figure 2A; 25–27 days).

### Upregulation of CXCL12 can effectively reduce demyelination in EAE

**FIGURE 3:**
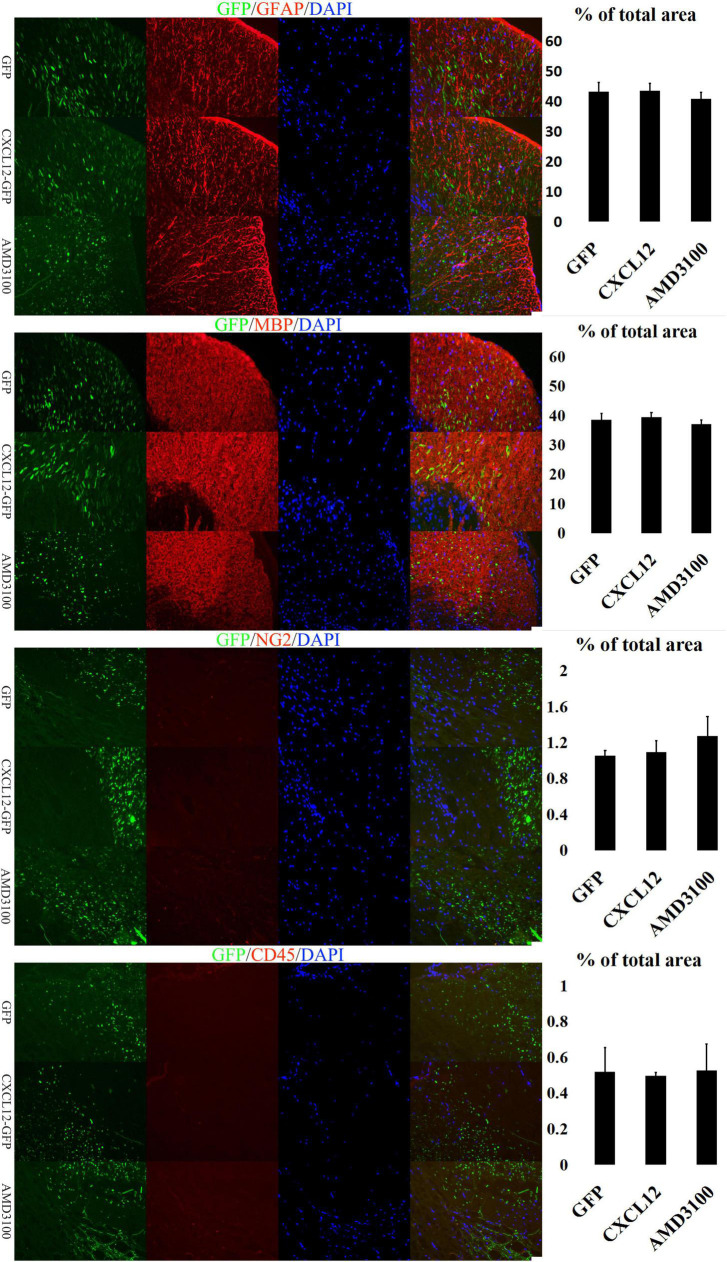
Upregulation of CXCL12 did not induced peripheral immune cell infiltration in the spinal cord at the initial stage. At the initiation stage of experimental autoimmune encephalomyelitis (EAE), the expression of glial fibrillary acidic protein (GFAP) and myelin basic protein (MBP) was similar in all three groups, indicating that the myelin sheath was intact. During this period, the OPC (NG2 +) and leukocyte (CD45 +) signals in the spinal cord were low. The bar charts show fluorescence intensity. Data presented as the mean ± SEM. “*” represents value of *P* < 0.05, “**” represents value of *P* < 0.01, “***” represents value of *P* < 0.001 by 2-tailed Student’s *t*-test or 1-way ANOVA, *n* = 4 rats. The bar in the lower right represents 50 μm.

Glia cells in the CNS, including microglia, astrocytes and oligodencrocytes, are very important for homeostasis maintaining and involved in the pathogenesis of MS directly (Stavropoulos et al., 2021). Oligodencrocytes are responsible for generating myelin sheaths and white matter tracts (Nave and Werner, 2014), and crosstalk between astrocytes and oligodendrocytes is closely related to the progress of MS (Patel et al., 2010; Stavropoulos et al., 2021). The immunofluorescence assay results revealed no significant differences in the expression levels of Myelin Basic Protein (MBP, a marker of mature oligodendrocytes) and Glial Fibrillary Acidic Protein (GFAP, a marker of astrocytes) among the three groups at the initial (Figure 3) and recovery stages (Figure 5) of EAE. However, at the peak stage of EAE, the fluorescence signals of MBP and GFAP in the spinal cord of the CXCL12 group were higher than those in the eGFP and CXCL12 + AMD3100 groups. The above immunofluorescence results are consistent with LFB-PAS staining results. Taken together, the results indicate that upregulation of CXCL12 can effectively alleviate the symptoms of EAE by reducing myelin damage.

**FIGURE 4:**
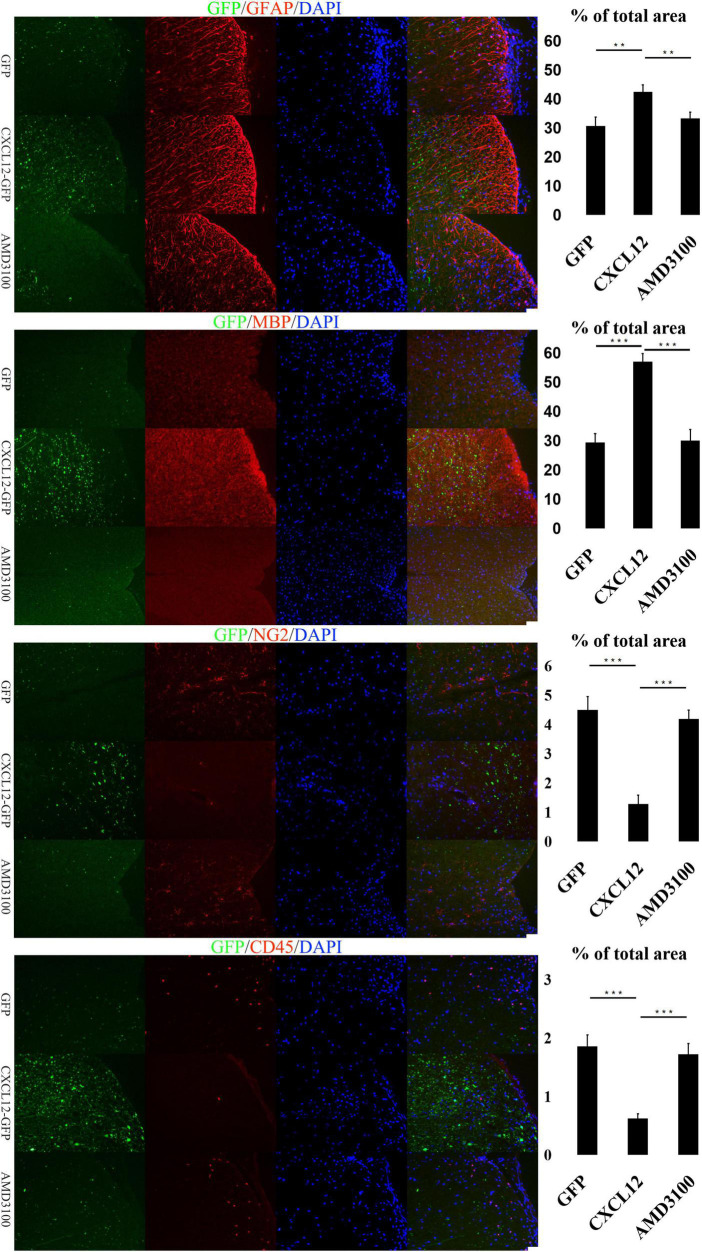
Upregulation of CXCL12 alleviated the spinal cord aberrations of experimental autoimmune encephalomyelitis (EAE) at the peak stage. At the peak stage of EAE, the significant differences in the glial fibrillary acidic protein (GFAP) and myelin basic protein (MBP) signals between the three groups showed that there was severe demyelination in the eGFP group and CXCL12-AMD3100 group. There were also significant differences in the CD45 and NG2 signals, suggesting that leukocyte infiltration likely led to deterioration of demyelination and that oligodendrocyte precursor cells (OPCs) were actively involved in remyelination. Data presented as the mean ± SEM. “*” represents value of *P* < 0.05, “**” represents value of *P* < 0.01, “***” represents value of *P* < 0.001 by two-tailed Student’s *t*-test or one-way ANOVA, *n* = 4–6 rats. The bar in the lower right represents 50 μm.

**FIGURE 5:**
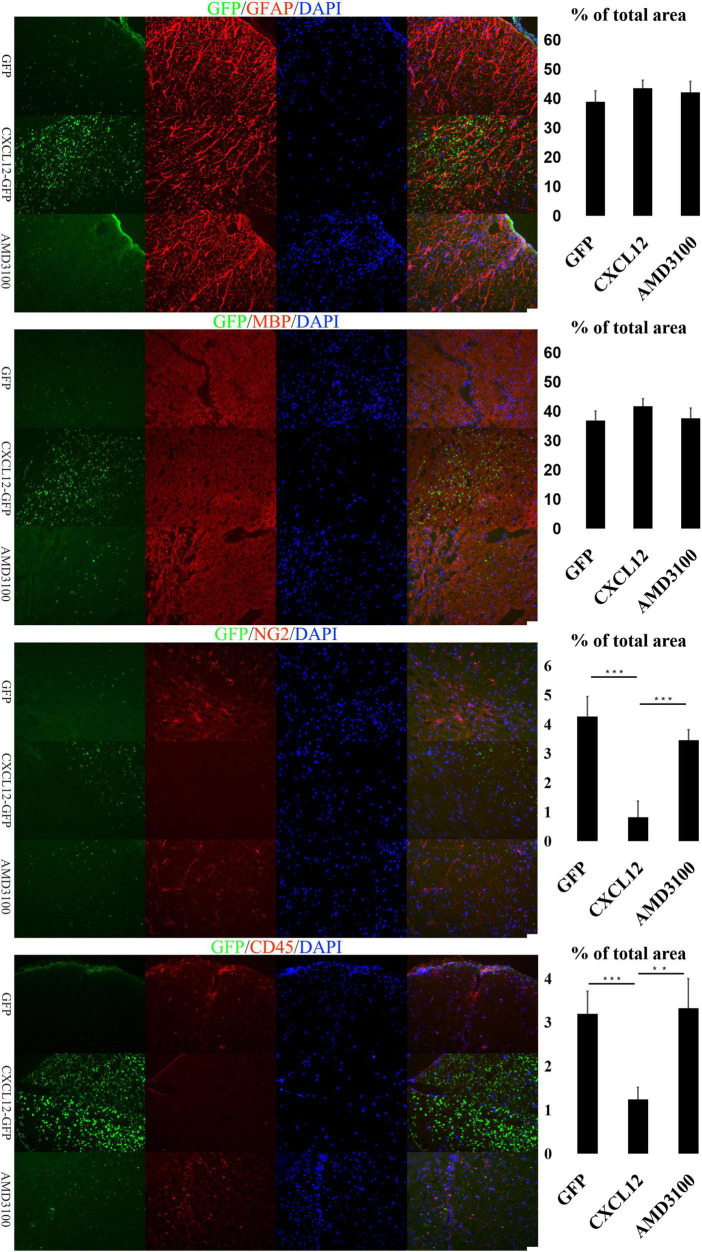
At the recovery stage of experimental autoimmune encephalomyelitis (EAE), upregulation of CXCL12 promoted the remyelination of spinal cord. During the recovery stage of EAE, the glial fibrillary acidic protein (GFAP) and myelin basic protein (MBP) signals indicated that the spinal myelin sheath was restored in the eGFP group and CXCL12 + AMD3100 group, but the infiltration of leukocytes (CD45 +) and the migration of OPCs (NG2 +) were still stronger than those in the CXCL12 group. Data presented as the mean ± SEM. “*” represents value of *P* < 0.05, “**” represents value of *P* < 0.01, “***” represents value of *P* < 0.001 by two-tailed Student’s *t*-test or one-way ANOVA, *n* = 4–6 rats. The bar in the lower right represents 50 μm.

The infiltration of peripheral immune cell into the CNS are the early events in EAE development, and are also observed in brains of MS patients (Floris et al., 2004; Ortiz et al., 2014). To determine the underlying mechanisms of CXCL12’s effects on the myelin sheath in the CNS, the signal of CD45 (a common leukocyte antigen) was detected in the three stages of EAE. At the initial stage, there were no significant differences in the CD45 signal among the three groups (Figure 3). However, during the peak stage and recovery stage, the CD45 signal in the CXCL12 group was significantly lower than that in the other groups (Figures 4, 5). Thus, there was less leukocyte infiltration in the CXCL12 group (Figures 3–5), which might be an important mechanism underlying the alleviation of the symptoms of EAE since lower leukocyte infiltration usually indicates reduced neuroinflammation.

### CXCL12 upregulation promotes the differentiation of OPCs into oligodendrocytes

In the CNS, Oligodorocytes is differentiated from OPCs. As shown in Figures 3, 4, NG2 (a biomarker of OPCs) was significantly upregulated in the spinal cord in the peak stage of EAE in the eGFP and CXCL12 + AMD3100 groups; the same trend was also observed in the recovery stage. This finding indicates that OPCs are actively involved in remyelination of the myelin sheath. However, in the CXCL12 group, it was difficult to detect OPCs at all stages of EAE, and the duration of the peak time (from the 13th to 22nd day) in the CXCL12 group was shorter than that of the other two groups (13 days on average), which might be because the upregulated CXCL12 promoted the mature differentiation of OPCs into oligodendrocytes, thus promoting regeneration of the spinal myelin sheath.

**FIGURE 6:**
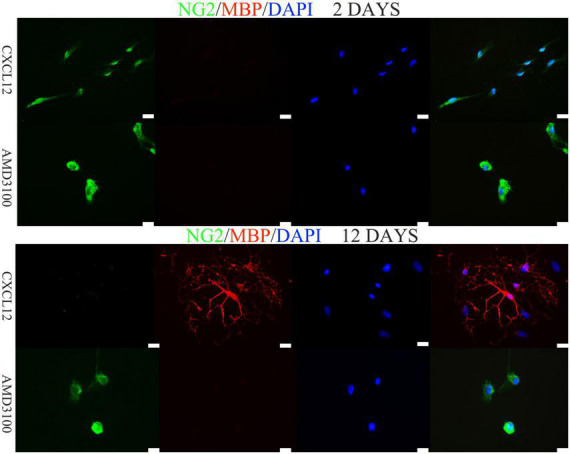
Differentiation of oligodendrocyte precursor cells (OPCs) into oligodendrocytes *in vitro* requires the induction of CXCL12. *In vitro*, 10 ng/ml CXCL12 promoted the differentiation of OPCs. Moreover, 400 ng/ml AMD3100 combined with CXCL12 inhibited differentiation. The bar in the lower right represents 50 μm.

**FIGURE 7:**
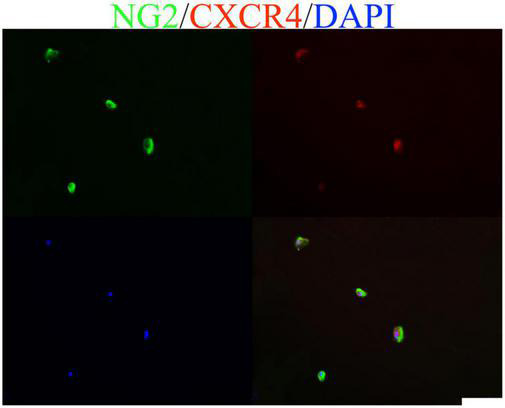
Oligodendrocyte precursor cells (OPCs) cultured *in vitro* coexpressed CXCL12 and its receptor CXCR4. The bar in the lower right represents 50 μm.

*In vitro*, OPCs of WISTAR rats were cultured in differentiation medium with either 0 or 10 ng/ml CXCL12. Exogenous CXCL12 (10 ng/ml) promoted the differentiation of OPCs into oligodendrocytes, as shown by immunostaining for MBP (Figure 6). AMD3100 was added to OPCs cultured in differentiation medium in the presence of CXCL12 (10 ng/ml). After 12 days, compared to the control differentiation culture (10 ng/ml CXCL12), AMD3100 administration strongly inhibited the differentiation of OPCs (Figure 6). Moreover, NG2 and CXCR4 coexpression in OPCs was assessed (Figure 7). The results were consistent with previous studies suggesting that the CXCL12/CXCR4 axis promotes the differentiation of OPCs *in vitro* and *in vivo* (Patel et al., 2010, 2012; Zilkha-Falb et al., 2016).

## Discussion

Oligodendrocyte precursor cells (OPCs) originate in subventricular zones that region is distant from white matter areas within the CNS. The migration, proliferation, and differentiation of OPCs are essential for repairment of demyelinated lesions. Therefore, the expression of chemoattractant which regulate neural precursor cells migrate and mature to replace damaged cells is essential for CNS injury response. CXCL12 and its receptors are widely expressed in the CNS and play an important role in the differentiation and maturation of NPCs and OPCs (Chu et al., 2017). Several previous studies have shown that CXCL12 can promote the migration of leukocytes to the lesion area of demyelination in the CNS during the progression of MS, thus aggravating the disease (Williams et al., 2014). In contrast, CXCL12 also promotes the migration and differentiation of OPCs to the lesion, thereby enhancing remyelination (Patel et al., 2010, 2012; Zilkha-Falb et al., 2016). In summary, the role of CXCL12 in the onset and progression of MS is complicated.

In this study, we up-regulated CXCL12 in the lumbar enlargement of spinal cord by AAV. This specific region was selected because the lesions of EAE predominantly distributed in the lumbosacral region of the spinal cord (Gibson-Corley et al., 2016). Immunofluorescence detection showed that the region of upregulated CXCL12 was limited to the vicinity of the PE-10 orifice. Immunofluorescence suggested that the level of CXCL12 in this region of the spinal cord was significantly higher in the CXCL12-GFP group than in the eGFP group (Figure 1A), and WB of SCH also confirm that (Figure 1B). Upregulated CXCL12 effectively alleviated the symptoms of EAE, reduced the clinical scores, and inhibited recurrence between days 24 and 30 in the EAE model. The MBP and GFAP signals in the CXCL12 group were stronger than those in the eGFP group at the peak stage of EAE. Moreover, a high level of CXCL12 did not induce significant leukocyte infiltration into the white matter of the spinal cord. Taken together with the results of the CXCL12 + AMD3100 group, these findings suggest the effect of CXCL12 can alleviate the symptoms at peak stage of EAE and inhibited the relapse. However, since the high level of CXCL12 did not induce more leukocyte infiltration, CXCL12 overexpression may not be a necessary or sufficient condition to induce leukocyte migration into the CNS.

Previous studies have shown that remyelination in rodents is closely related to OPCs migration and differentiation (Lombardi et al., 2019). At the peak stage of the EAE model, the level of CXCL12 was upregulated in the spinal cord; this is consistent with several studies that have shown that CXCL12 enhanced the process of remyelination (Zeis et al., 2018; Gao et al., 2019; Beigi et al., 2020). Furthermore, the findings of the current study indicated that the NG2 signal (OPCs) in the spinal cord of the CXCL12 group was significantly lower than that of the other two groups at the peak stage and recovery stage, while the MBP signal was much stronger, suggesting that the differentiation of OPCs into oligodendrocytes in the CXCL12 group was due to the continuously upregulated CXCL12. *In vitro*, CXCL12 (10 ng/ml) promoted the differentiation of OPCs into oligodendrocytes (Figure 6), and the CXCR4 antagonist AMD3100 strongly inhibited the differentiation of OPCs (Figure 6). This finding suggests that CXCL12 protects the CNS of rats by promoting the differentiation of OPCs into oligodendrocytes.

The clinical scores of the CXCL12 group were significantly lower than those of the eGFP group and the AMD3100 treatment group, and there was no recurrence during days 24–30. *In vitro*, 10 ng/ml CXCL12 promoted the differentiation of OPCs into oligodendrocytes, which is consistent with previous studies (Zilkha-Falb et al., 2016). These results suggest that upregulation of CXCL12 can alleviate EAE symptoms and inhibit EAE recurrence through the underlying mechanism of initiating OPC maturation and differentiation. No significant difference in leukocyte infiltration in the EAE initial phase was found between the CXCL12 group and the other two groups using CD45 detection. At the EAE peak stage, the CXCL12 group had a significantly lower D45 + signal than the other groups. Yamasaki et al. (2014) reported that infiltrating monocyte-derived macrophages initiate demyelination; therefore, less infiltration of macrophages may delay the onset of EAE and alleviate the symptoms of EAE in the peak stage. *In vivo*, several previous studies have reported that CXCL12 acts as a T-cell chemoattractant at low doses and a chemorepellent at high doses (Poznansky et al., 2000; Vianello et al., 2006). Furthermore, Meiron et al. (2008) demonstrated that CXCL12 could transform effector Type 1 T helper (Th1) cells in the CNS into regulatory T cells that produce interleukin (IL)-10, an anti-inflammatory cytokine, thereby reducing neuroinflammation and further reducing the infiltration of leukocytes. This observation may explain why the infiltration of leukocytes was lower in the CXCL12 group than in the other groups.

The data presented in this study indicate that upregulation of CXCL12 in in the lumbar enlargement of the spinal cord can significantly reduce the symptoms and recurrence of EAE in a rat model. Furthermore, the CXCL12/CXCR4 axis may enhance remyelination by promoting the differentiation and maturation of OPCs, suggesting that the CXCL12/CXCR4 axis could be a promising therapeutic candidate to improve remyelination in MS.

## Data availability statement

The original contributions presented in this study are included in the article/supplementary material, further inquiries can be directed to the corresponding authors.

## Ethics statement

This animal study was reviewed and approved by the Ethics Committee of the Chinese PLA General Hospital.

## Author contributions

DL, HJL, HS, and BC performed the experiments. DL, HJL, HS, and SW wrote the original manuscript. DL, JF, MS, HZ, WB, and HEL revised the manuscript. All authors contributed to the article and approved the submitted version.

## Footnotes

1
https://www.addgene.org/protocols/aav-titration-qpcr-using-sybr-green-technology/
